# Obesity as a Prognostic Factor of Central Nervous System Relapse in Children with Acute Lymphoblastic Leukemia: A Single-Centre Study and Literature Review

**DOI:** 10.1155/2022/7783823

**Published:** 2022-03-21

**Authors:** Guo-qian He, Yi-ling Dai, Ming-yan Jiang, Ju Gao, Xia Guo

**Affiliations:** ^1^Key Laboratory of Birth Defects and Related Diseases of Women and Children, Ministry of Education, West China Second University Hospital, Sichuan University, Chengdu, Sichuan 610041, China; ^2^Department of Pediatrics, West China Second University Hospital, Sichuan University, Chengdu, Sichuan 610041, China

## Abstract

Relapse as the commonest treatment failure through chemotherapy of child presented with acute lymphoblastic leukemia (ALL) is usually within 3 years of remission. Central nervous system (CNS) is expected as a site of extramedullary relapse in 3–8% of child leukemia, often leading to a poor prognosis. A few patients may have headache and vomiting and can be diagnosed without difficulty. However, most patients present with asymptomatic conditions. Obesity has become one of the greatest reported complications of children ALL survivors. Rarely, obesity presentation can be the first manifestation of CNS leukemia. Here, we present three unusual cases with B-ALL presentation of obesity as the first symptom at the time of CNS relapse after achieving remission. This highly localized presentation is unusual and would hopefully inform clinicians to have a high index of suspicion for relapse in children with ALL.

## 1. Introduction

Acute lymphoblastic leukemia (ALL) is the most common pediatric malignancy, with an overall survival rate of 90% under current improved risk-directed treatment based on cytogenetic aberrations, molecular characterization, and immunophenotyping [1]. However, pediatric ALL has a tendency to relapse, with a relapse rate of 10–20% within 3 years of remission [1, 2]. The relapse of ALL remains a negative prognostic factor, even with aggressive salvage strategies [2, 3]. Relapsed ALL is thought to be distributed widely in the bone marrow. Extramedullary relapses are unusual, and approximately 3–8% of children with ALL suffer from relapse in the central nervous system (CNS). Children suffering from CNS leukemia (CNSL) are believed to have poorer outcomes [4, 5]. The most common form of CNSL is meningeal metastasis. The conventional method for diagnosing CNSL is dependent on clinical manifestations, imaging manifestations, and primarily microscopic examination of cerebrospinal fluid (CSF) [4, 6]. However, CNSL manifestations can vary. In most cases, patients do not have any neurological signs or symptoms. Only a few patients present with headache and vomiting [7, 8].

Obesity is a complex, multifactorial disease and an acknowledged late effect in childhood ALL survivors [7, 9–12] [8]. Among all survivors, an increased risk of becoming obese results in substantial physical and psychosocial morbidity and further complicates other therapy-associated adverse effects, such as infections, osteopenia, and osteonecrosis. Cranial radiotherapy (CRT) has also been reported to be associated with the incidence of obesity in childhood ALL, which can cause hypothalamic-pituitary dysfunction. However, obesity is rarely present at ALL relapse. Despite reports of obesity as a very rare manifestation of CNS relapse in patients with leukemia, the exact pathogenesis is not clear[7, 8, 11, 13]. There remains little medical literature reporting hypothalamic obesity syndrome or Cushing syndrome related to leukemic infiltration of the CNS.

Here, we report three unusual cases in which ALL patients presented with obesity at the time of CNS relapse to highlight (1) a rare presentation with obesity but no signs of disease in common central nervous system sites and (2) the potential for ALL to relapse after achieving remission. We also describe the children's response to intensive chemotherapy and discuss the possible mechanisms of CNSL-induced obesity based on a literature review. A high index of suspicion for CNS relapse should be kept in mind in children with ALL who manifest obesity.

## 2. Case Series

### 2.1. Definition of Obesity

Clinical measures of weight and height were recorded to the nearest 0.1 kg and 0.1 cm, respectively. We used obesity definitions in accordance with the China child growth standards. Overweight was defined as a body mass index (BMI) ≥ the 85th percentile and < the 95th percentile for age and sex. Obesity was defined as body mass index (BMI) ≥ the 95th percentile for age and sex [14].

### 2.2. Dataset

We retrospectively analyzed the medical records of ALL patients with CNS leukemia relapse treated in the pediatrics blood and oncology center of West China Second University Hospital between 2015 and 2020. Patients presenting with obesity at relapse were included. Patients with obesity or CNS leukemia at diagnosis were excluded. All patients were treated with the China children cancer group protocol (CCCG-ALL-2015), modified from St. Jude Children's Research Hospital Total-XV protocol for newly diagnosed patients with ALL [15, 16]. In these protocols, based on the age, the white blood cell (WBC) count at diagnosis, morphology, immunophenotype, cytogenetic aberrations, molecular characterization criteria, fusion genes, and the bone marrow response at Day 19 and Day 46 as monitored by bone marrow (BM) biopsy and minimal residual disease (MRD), the patients were stratified into three different risk groups (low-risk (LR) group, intermediate-risk (IR) group, and high-risk (HR) group). This estimated risk guided the intensity of the treatment.

### 2.3. Epidemiological Characteristics of Patients at Relapse

A total of 3 patients with CNS relapse presenting with obesity were included. The three patients' clinical characteristics are summarized in Table 1. All patients were male. The median age at diagnosis was 3.53 years (range 2.0–4.10). The median age at relapse was 5.3 years (range 2.5–7.7), and the median follow-up time from relapse was 29.7 months (range 23.6–33.8 months). The three patients were characterized by fusion gene negativity and had no hyperleukocytosis at diagnosis. Two patients were diagnosed with precursor B-cell ALL (B-ALL) with normal chromosomes, and 1 patient was diagnosed with B-cell ALL but was positive for hyperdiploidy. There was no evidence of CNS involvement at the time of diagnosis. Only one patient had a known history of *α*-thalassemia and primary nephritic syndrome.

The 3 patients were determined to have LR status and were started on chemotherapy with the CCCG-ALL-2015 protocol. Total CCCG-ALL-2015 therapy for the low-risk group lasted approximately 2.5–3.0 years and consisted of 3 treatment phases: induction (7 weeks), consolidation (8 weeks), and continuation (reinduction I and reinduction II, 110 weeks). All patients received dexamethasone at a dose of 6 mg/m^2^/day on Days 1 to 4 and prednisone at a dose of 45 mg/m^2^/day for 24 days during induction, and they received dexamethasone at a dose of 8 mg/m^2^/day on Days 1 to 7 and Days 15–21 during reinduction I (continuation week 16–31) and on Days 1 to 7 during reinduction II (week 32–34). Seven-day dexamethasone pulses (every 4 weeks) were administered during continuation therapy at a dose of 8 mg/m^2^/day for patients with LR status from continuation weeks 16 to 109.

Depending on their risk and CNS status at diagnosis, they received prophylactic CNS-directed therapy involving triple intrathecal chemotherapy with dexamethasone (3 mg/time per dose), cytosine arabinoside, and methotrexate. Two patients received CNS prophylaxis with intrathecal (IT) methotrexate (capped at 12.5 mg in patients 3 years of age) for 2 doses in the induction stage and a total of 18 doses as per the protocol. One patient had a lumbar puncture injury in the first IT administration (capped at 9 mg in patients 2 years of age) and received 5 doses in the induction stage and a total of 21 doses. None of the patients were given cranial irradiation. All patients' bone marrow status showed continuous CR for 2.0–2.10 years. There was no evidence of CNS involvement in any of the phases.

### 2.4. Obesity at Diagnosis and Relapse

Prior to admission, while the 3 patients were in the maintenance phase of chemotherapy, they presented to the pediatric clinic with complaints of excessive weight gain for 2–3 months, with an increased appetite. There was no family history of endocrine disorder or parental consanguinity.

**Figure 1:**
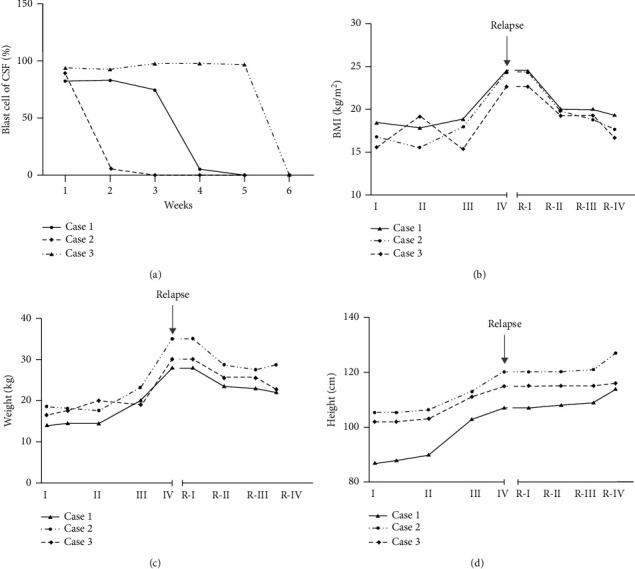
Note that the blast in CSF, BMI, weight, and height at diagnosis and at the routine clinic visits in the phase of ALL chemotherapy. I the start of induction and the end of induction according to CCCG-ALL-2015, II: the start of consolidation according to CCCG-ALL-2015, III: the start of continuation-I according to CCCG-ALL-2015. IV: the start of continuation-II according to CCCG-ALL-2015. R–I: the start of induction according to CCCG-ALL-2017 relapse, R–II: the start of consolidation-I according to CCCG-ALL-2017 relapse, R–III: the start of consolidation –II according to CCCG-ALL-2017 relapse, R–IV: the start of maintenance-II according to CCCG-ALL-2017 relapse. Clinical measures of weight and height were recorded to 0.1 kg and 0.1 cm, respectively.

Weight and height data were obtained from routine clinic visits at our hospital at 5 time points: the start of induction (week 1), the end of induction (week 7), the start of reinduction I (week 16), the start of reinduction II (week 35), and the relapse time. As shown in Figure 1, the three patients in our center had no obesity at diagnosis or at the routine clinic visits during ALL chemotherapy. However, we found that one patient in our center was overweight at diagnosis, and two patients were persistently overweight during treatment according to the children's BMI standards. Moreover, 2 of the patients experienced reduced activity and drowsiness in the daytime, 1 patient experienced emotional changes, and 1 patient even experienced polyuria and polydipsia.

On examination, the 3 patients had characteristic initial symptoms with central obesity, buffalo hump and moon facies but normal neurological examination findings. Of these, only 1 patient had hypertension (137/92 mm Hg). For the differential diagnosis of obesity, endocrine studies were performed. As shown in Table 2, laboratory tests revealed normal complete blood count, fasting blood glucose, serum fasting insulin, oral glucose tolerance test, C peptide-insulin releasing test, thyroid function, serum insulin, blood lipid, adrenaline, norepinephrine, sex hormone, and routine urine test results. Endocrine study findings suggested that only one patient in our center had elevated levels of morning ACTH, morning serum cortisol at 8 a.m. (1415.2 nmol/L, normal up to 618 nmol/L), and serum cortisol at 2 p.m. (515.7 nmol/L, normal up to 460 nmol/L) and an abnormal diurnal rhythm of cortisol. The serum aldosterone (42.59 ng/ml), ACTH (55.40 pg/ml), and 24-h urinary-free cortisol (215.0 ng/ml) levels were slightly elevated. This loss of diurnal variation strongly suggested Cushing syndrome. However, morning cortisol levels (39.4 nmol/L) and ACTH levels (6.09 pg/ml) were decreased after an overnight single dose (0.5 mg) of dexamethasone. CT of the abdomen showed local nodular thickening in the left adrenal.

### 2.5. CNS Status at Relapse

In our study, one patient experienced facial paralysis, and the other two patients had no characteristic CNS symptoms. Peripheral blood counts were available for the 3 patients with relapse in our center. Cytological examination of CSF was the most conventional examination for diagnosing CNSL. In this study, the examination of CSF revealed blast cells suggestive of CNS relapse. Flow cytometry of CSF was then performed for a detailed CNSL diagnosis [4]. In our study, aberrant expression of CD19-positive B cells and leukemic blasts was identified based on their aberrant expression of CD10, CD20, and CD34 in the absence of CD3. The three patients were found to have a significantly high relapse risk, specifically for B-ALL, by flow cytometry.

In this study, no edema, infiltration, brain parenchymal or meningeal enhancement, or pituitary adenoma was detected by CT or magnetic resonance imaging (MRI) of the brain in any patient. In one patient, CT of the brain showed several high-density lesions in the left frontoparietal cortex suggestive of possible intracranial hemorrhage. MRI of the brain in one patient showed a high signal in the posterior pituitary. MRI of the brain in one patient showed mild subdural effusion, bilateral lateral ventricle widening, and a slight small volume of the pituitary gland (3 mm) accompanied by an empty sella.

Moreover, 2 patients had combined CNS and BM relapse. Bone marrow aspirate revealed >5% leukemic cells. Bone marrow MRD was elevated. We diagnosed the two patients with CNS relapse combined with BM relapse based on the above findings.

### 2.6. Treatment Response

In our center, the three patients were treated with the CCCG-ALL-relapse-2017 protocol, which was modified from a UK Children's Research protocol for first relapse patients with ALL (ALL R3 trial) [1, 17]. According to the protocol for children with a first relapse of ALL, the clinical and laboratory characteristics were used to stratify the patients according to their risk of relapse (standard-risk group, intermediate-risk group, and high-risk group). According to the CCCG-ALL-relapse-2017 protocol, the three patients experienced relapses >18 months and <36 months from diagnosis and were thus stratified into the intermediate-risk group. These three CNS-positive patients (CNS 3) were then treated with triple intrathecal chemotherapy each week. Notably, after relapse, minimal residual disease measurements might be the only prognostic marker to predict survival after chemotherapy reinduction.

During the follow-up, 2 patients developed epileptoid seizures, intracranial hypertension syndrome and central respiratory failure after starting induction therapy, but one patient's electroencephalograms revealed abnormal school-age awake EEGs. Two patients developed paresthesia, such as a lack of superficial reflexes and the presence of formication. Moreover, two patients developed urinary difficulty or incontinence. One patient developed blurred vision and photism in the left eye, and examination revealed left optic nerve atrophy with visual evoked potentials. No pituitary adenoma or abnormal optic nerve was detected. One patient developed decreased lower limb muscle strength. MRI of the hip joint showed fatty changes in the bilateral musculus gluteus maximus. Electromyography of the limbs showed changes in lower limb abnormal somatosensory evoked potentials and peripheral nervous function.

In follow-up evaluations, all three patients responded well to chemotherapy in the first stage of the treatment, accompanied by reduced blasts in the CSF (Figure 1) and BM sustained remission with MRD negativity (Table 3). As shown in Figure 1, blasts in CSF (blast %) on Days 0, 7, 14, 21, and 28 of induction were decreased. Obesity showed dramatic improvement following the initiation of chemotherapy. Our patients showed a rapid decrease in weight and BMI following consolidation therapy and maintained their weight during the maintenance therapy. To date, cranial or cranio-spinal irradiation has not been used. One patient experienced a second CNS relapse, as identified in CSF cytology. The parents subsequently chose palliative treatment for their son, but he died.

## 3. Discussion

In most studies, obesity is a reported complication of childhood acute lymphoblastic leukemia but rarely presents as the first manifestation of CNS relapse. Therefore, it seems important to distinguish CNSL from other causes of obesity and to identify the key warning factors for CNSL. Moreover, the etiology of obesity in CNSL of ALL is not yet fully understood. There is still no explicit predictor for CNS relapse. Next, we discuss the possible mechanisms of CNSL-induced obesity based on a literature review.

We searched for articles describing cases of CNS relapse of ALL in children using PubMed, the Web of Science and three major journal databases in China, Wanfang Data, VIP, and CNKI Data, with the search term “obesity” from January 2000 to January 2021. Cases were accepted if the reports provided sufficient clinical features of obesity, CNSL and endo relapse diagnosis in child ALL. Cases of malignancy as defined by the original authors were accepted without verification. Duplicate case reports were excluded. Overall, 4 cases from the English literature were included. Patient characteristics are detailed in Tables 2 and 4. Of these, all patients presented with obesity in the maintenance phase of chemotherapy. Two patients were female, and 2 patients were male. All patients had B-cell or pre-B-cell ALL. There were no patients with CNS involvement at diagnosis in the reported literature. However, one patient did not receive CNS prophylaxis in the form of intrathecal injection, while the remaining patients were treated with systemic and intrathecal chemotherapy alone, and one patient also received radiation treatment for CNS prophylaxis. Of these, 3 patients had high ACTH and serum cortisol. Three patients did not receive therapy due to disease progression or family factors.

The diagnosis of CNSL is dependent more on clinical manifestations, imaging manifestations, and primarily the cytological examination of CSF. However, CNSL manifestations can vary, and some patients are asymptomatic. Seven patients (3 patients in our center and 4 patients in the literature) had no characteristic CNS symptoms, except central obesity along with hyperphagia and cushingoid facies as initial symptoms. Five patients experienced reduced activity, and 6 patients experienced emotional changes. In our center, only one patient experienced facial paralysis. This may indicate that patients undergo changes in their routine lifestyle: increased energy intake and reduced habitual physical activity. Moreover, one patient in the literature was reported to have experienced headache. Moreover, ALL children with hyperleukocytosis at diagnosis, BCR-ABL1, MLL rearrangement, hypodiploidy (less than 45 chromosomes), TCF3-PBX1 fusion, or a diagnostic traumatic lumbar puncture are considered to be at increased risk for CNS relapses [6, 18]. However, we found that the 3 patients in our center did not significantly differ in these features.

Nevertheless, the etiology of obesity in CNS relapse of ALL is not yet fully understood. Next, we discuss the possible mechanisms of CNSL-induced obesity based on a literature review. (i**)** CNSL is caused by the infiltration of leukemia cells into the CNS. The most common form of CNS involvement is meningeal metastasis. Infrequently, CNS relapse may occur in the form of cerebral parenchymal mass lesions and posterior reversible encephalopathy syndrome. Leukemic infiltration of the pituitary gland is extremely rare [7, 19]. ACTH-dependent cushing syndrome (CS) was reported to be due to leukemic infiltration of the pituitary gland. Leukemia inhibitory factor (LIF), as an inhibitor of mouse myeloid leukemia cells, could be a possible causative factor and the cause of this syndrome [7, 11]. In the literature, three patients were diagnosed with ACTH-dependent endogenous cushing syndrome during the maintenance phase of chemotherapy for ALL. However, there was no direct evidence. (ii) Hypothalamic obesity syndrome most commonly manifests with hyperphagia and intractable weight gain following hypothalamic injury due to surgical intervention, cranial irradiation or CNS tumor resection [10, 13, 20–22]. This syndrome is rare at diagnosis in childhood cancer but has been reported with CNS relapse of ALL and lymphoblastic lymphoma [8, 10]. Three different mechanisms of CNSL-induced obesity associated with hypothalamic dysfunctions have been described in the literature: a dysfunction of the ventromedial hypothalamus, a dysfunction of the outputs of the paraventricular nucleus of the hypothalamus and damage to the ventromedial hypothalamus (VMH). In our patients, although there were abnormal findings on pituitary gland MRI or CSF examination, no ACTH hypersecretion was found in the serum, and the boys were determined to have hypothalamic obesity rather than Cushing syndrome.

Among our patients, two boys had no hypothalamic obesity syndrome or Cushing syndrome and excessive ACTH secretion. However, the boys were overweight at diagnosis or at routine clinic visits in the ALL chemotherapy phase. Many studies have shown that obesity is among the medical problems in survivors of childhood ALL. These obese patients are at risk of metabolic syndrome (MS) [9, 23]. MS may exist throughout the whole treatment process and is associated with the BMI *Z-*score, the chemotherapy pharmacokinetics of overweight patients and the antiapoptotic effects in leukemic cells caused by adipocytes [12, 14, 23]. When dosing is based on body-surface area, obese patients receive lower doses of drug per kilogram of weight and are at higher risk of relapse. However, the two boys did not have elevated plasma glucose, dyslipidemia, hypertension, or a prothrombotic and proinflammatory state. Therefore, more research is needed to support this hypothesis.

In summary, our study showed that obesity can be considered an independent predictor of CNS relapse risk, mainly in low-risk (LR) groups of children. These results could be explained by Cushing syndrome or hypothalamic obesity syndrome changes in obesity patients and by the antiapoptotic effects in leukemic cells caused by adipocytes. Our study aims to highlight the need for CSF evaluation in patients presenting with obesity to evaluate the possibility of underlying CNS leukemia. More importantly, measurements of weight, BMI, and body fat percentage are suggested for evaluation at every follow-up visit.

## Figures and Tables

**Table 1:** Clinical and laboratorial characteristics of patients at diagnosis and at relapse.

Characteristic	No. of patients n	
Total	**3**	

*Gender*		
Male	3	
Female	None	

*Median age at diagnosis (years)*	3.5 ± 1.3	
*Median age at relapse (years)*	6.2 ± 1.9	

*Phenotype*		
B	3	
T	None	

*Cytogenetic features*		
BCR-ABL1	None	
TCF3/PBX1	None	
ETV6/RUNX1	None	
KMT2A-r	None	
Hyperdiploidy	1	
Others	None	

*WBC count at diagnosis*		
＞50 × 109/L	None	
≤50 × 109/L	3	

*WBC count at relapse*		
＞50 × 10^9^/L	None	
≤50 × 109/L	3	

*CNS status at diagnosis*		
CNS 1	3	
CNS 2	None	
CNS 3	None	

*IT chemotherapy in first course*		
Yes	3	
No	None	

*Blast in PB at the first IT*		
Yes	0	
No	3	

*Traumatic lumbar puncture in the initial IT*		
Yes	1	
No	2	

*Day 19 of BM biopsy*		
M1	2	
M2	1	
M3	None	

*Day 19 MRD of BM*		
<1%	3	
≥1%	None	

*Day 46 of BM biopsy*		
M1	1	
M2	2	
M3	None	

*Day 46 MRD of BM*		
<1%	3	
≥1%	None	

*Risk at diagnosis according CCCG-ALL-2015*		
LR	3	
IR	None	
HR	None	

*Prior remission status*		CR
*Time to relapse (moths)*		
Very early(<18)	None	
Early(18–36)	3	
Late(>36)	None	

*Relapse site*		
Isolated CNS	1	
CNS + BM	2	
Isolated BM	None	

*CNS status at relapse*		
CNS 1	None	
CNS 2	None	
CNS 3	3	

*Flow cytometric of CNS at relapse*		
Yes	3	
NO	0	

*Risk at relapse according to [_CCCG_ALL_relapse_2017]*		
LR	0	
IR	3	
HR	0	

B, B-cell precursor ALL; *T*, T-cell ALL; BM, bone marrow; CNS, central nervous system; LR, low risk; IR, intermediate risk; HR, High risk; PB, peripheral blood; WBC, white blood cells; Bone marrow biopsy, M1: <5% blasts in the bone marrow, M2: 5–25% blasts, M3: >25% blasts in the bone marrow; MRD, minimal residual disease; CR, Complete remission; CNS status, CNS 1: no blasts cells, CNS 2: blasts <5 leukemic cells/µL; CNS 3: blasts ≥5 leukemic cells/µL, and/or clinical signs of CNS leukemia (cranial nerve palsy, intracranial mass on MRI, or retinal involvement). Irrespective of CNS status, lumbar punctures were classified either as traumatic (>10 erythrocytes/*μ*L) or nontraumatic. Negative MRD was defined as <0.01% leukemia detected in a bone marrow aspirate specimen. Complete remission (CR) was defined as <5% blasts in the bone marrow, no evidence of extramedullary disease, and recovery of peripheral counts with an absolute neutrophil count (ANC) ≥1000/µL and platelet count ≥100 × 109/L.

**Table 2:** Auxiliary examination at CNS relapse.

Features	CNSL cases, *n*	Reported in other studies, n
*Hematological abnormality*		
Pancytopenia	None	Data missing
Blast in PB	None	Data missing
Abnormal blood fat	None	Data missing
Abnormal of liver function	None	Data missing

*Endocrine examination*		
High ACTH	1	3
High serum cortisol	1	3
Abnormality thyroidal function	1	None
Abnormality OGTT	None	None
Abnormality insulin	None	None
Brain CT/MRI abnormality	2	3
Ultrasonography abnormality	1	Data missing

PB, peripheral blood.

**Table 3:** Response to therapy and outcomes in our center.

Patient	Bone marrow blasts pretreatment (%)	MRD pretreatment (%)	Response	MRD posttreatment	Second relapse
Case1	6.0	0.53	CR	＜0.01%	Yes
Case2	Data missing	＜0.01%	CR	＜0.01%	No
Case3	Data missing	4.65	CR	＜0.01%	No

CR, complete response; MRD, minimal residual disease.

**Table 4:** List of CNSL relapse studies of ALL reported with obesity.

		Studies		
		Jain^1^	Luo^2^	Sahin^3^
No. of patients		2	1	1
Age(years)		2.5–6.5	6	3.8
*Male/female*		1/1	1/0	0/1
*Definition(Weight centile)*		> 97th percentile	>97th percentile	Data missing
*Immunophenotype*		B Or pre B	Pre-B	B
*CNS invasion at diagnosis*		No	No	No
*Cytogenetic features*		Data missing	BCR-ABL1 negative	Data missing
Treatment at diagnosis				
Protocol	International network for cancer treatment and research protocol		Modified ALLIC BFM2002 protocol	ALL-BFM95 study protocol
CNS prophylaxis (IT)	1 received		Yes	Yes
Cranial irradiation	1 received		No	No
*Clinical data at relapse*	Maintenance chemotherapy		Maintenance chemotherapy	Maintenance chemotherapy
*Time to CNS relapse (years)*	1.1–1.5		2	1.5
*Management of CNS relapse*	No treatment		Received induction chemotherapy and IT every 3 days	No treatment
*Disease status*	1 Deceased 1 lost to follow-up		The CSF normalized after the third intrathecal injection	Deceased
*Note (possible mechanism)*	Cushing syndrome		Hypothalamic obesity	Cushing syndrome

B, B-cell precursor ALL; BM, bone marrow; CNS, central nervous system; IT, intrathecal injection.

## Data Availability

The data used to support the findings of this study are included within the article.

## Abbreviations

CNSL: Central nervous system leukemia
ALL: Acute lymphoblastic leukemia
CS: Cushing's syndrome
HO: Hypothalamic syndrome
MS: Metabolic syndrome.
